# Advances in cryo-electron tomography and subtomogram averaging and classification

**DOI:** 10.1016/j.sbi.2019.05.021

**Published:** 2019-10

**Authors:** Peijun Zhang

**Affiliations:** 1 Division of Structural Biology, Wellcome Trust Centre for Human Genetics, University of Oxford, Oxford, OX3 7BN, UK; 2 Electron Bio-Imaging Centre, Diamond Light Source, Harwell Science and Innovation Campus, Didcot OX11 0DE, UK; 3 Department of Structural Biology, University of Pittsburgh School of Medicine, Pittsburgh, PA 15260, USA

## Abstract

Cryo-electron tomography (cryoET) can provide 3D reconstructions, or tomograms, of pleomorphic objects such as organelles or cells in their close-to-native states. Subtomograms that contain repetitive structures can be further extracted and subjected to averaging and classification to improve resolution, and this process has become an emerging structural biology method referred to as cryoET subtomogram averaging and classification (cryoSTAC). Recent technical advances in cryoSTAC have had a profound impact on many fields in biology. Here, I review recent exciting work on several macromolecular assemblies demonstrating the power of cryoSTAC for *in situ* structure analysis and discuss challenges and future directions.

## Highlights

• Cryo-electron tomography (cryoET) subtomogram averaging has emerged as a structural biology method for sparse and heterogenerous sampls.
• CryoET subtomogram averaging enables *in situ* structure determination.
• CryoET subtomogram classification can delineate different conformational states of macromolecular complexes.
• Future developments in cryoET and correlative super resolution microscopy promises to bring unprecedented integration of cell biology and structural biology.

**Current Opinion in Structural Biology** 2019, **58**:249–258

This review comes from a themed issue on **Biophysical and computational methods**

Edited by **Mark Yeager**

For a complete overview see the Issue and the Editorial

Available online 5th July 2019

**https://doi.org/10.1016/j.sbi.2019.05.021**

0959-440X/© 2019 The Author. Published by Elsevier Ltd. This is an open access article under the CC BY license (http://creativecommons.org/licenses/by/4.0/).

## Introduction

Over the past five years, we have witnessed a huge leap in the field of cryoEM, in particular single-particle image analysis (SPA), with which structures of proteins and protein complexes are routinely determined to atomic and near-atomic resolutions [1,2]. It is considered the method of choice for determining the structures of large macromolecular assemblies, as it is more tolerant of structural heterogeneity and requires much less material than crystallographic methods. However, for pleomorphic and heterogeneous biological specimens that are not amenable to SPA, such as intact cells, organelles, pleomorphic viruses and variable macromolecular assemblies, cryo-electron tomography (cryoET) has been the method of choice [3]. In cryoET, a series of projection images from the same object are recorded as the sample is tilted to various angles relative to the incident electron beam. The images are subsequently aligned and reconstructed to generate a 3D tomogram. It provides a 3D volume of a single unique specimen without averaging. CryoET allows 3D imaging of frozen-hydrated biological specimens in a close to native state. Under optimal conditions, structural information to near-atomic resolution can be achieved. CryoET is a versatile technique that can be applied to a broad range of specimens, from isolated protein complexes to large eukaryotic cells [3].

Current practice of cryoET involves two main approaches, namely molecular cryoET and cellular cryoET. Molecular cryoET is typically employed to study *in vitro* purified ‘single-particle’ samples, often pleomorphic and not amenable for cryoEM SPA. This method has been excellent for generating initial models for cryoEM SPA, particularly when samples are relatively homogeneous. More recently, molecular cryoET has been applied to analyze repeating structures within larger pleomorphic objects using a process called cryoET subtomogram averaging and classification (cryoSTAC), when individual repeating units (i.e. subtomograms) are aligned in 3D and averaged to improve the signal-to-noise ratio (SNR) and the map resolution [4,5]. These 3D subtomograms can be further classified into multiple functional states or conformations [4,6]. Cellular cryoET, in contrast, has been applied to large pleomorphic objects such as intact bacteria and eukaryotic cells. It has been classically used for morphological analysis, until recently when high-resolution *in situ* structures of cellular complexes and assemblies have been obtained using cryoSTAC [7^••^]. Compared to SPA, cryoSTAC is arguably the greatest strength of cryoET, because each particle exists as a unique 3D reconstruction and allows for direct analysis of the 3D variance.

In recent years, advances in sample preparation, detector technology, phase plate imaging and image processing tools have enabled unprecedented characterization of protein complexes, *in situ* and *ex situ* [3]. CryoET and cryoSTAC have emerged as powerful methods for visualizing the molecular organization within a native cell or organelle, potentially allowing determination of protein complexes in their functional states and native environment to near-atomic resolution. In this review, I describe a typical cryoSTAC workflow and review advances during the past few years, focusing on high-resolution structure determination and classification of functional states. I highlight some exciting cases where near-atomic resolution has been achieved and novel functional insights have been obtained *in situ* via cryoSTAC.

## CryoSTAC workflow

**Figure 1:**
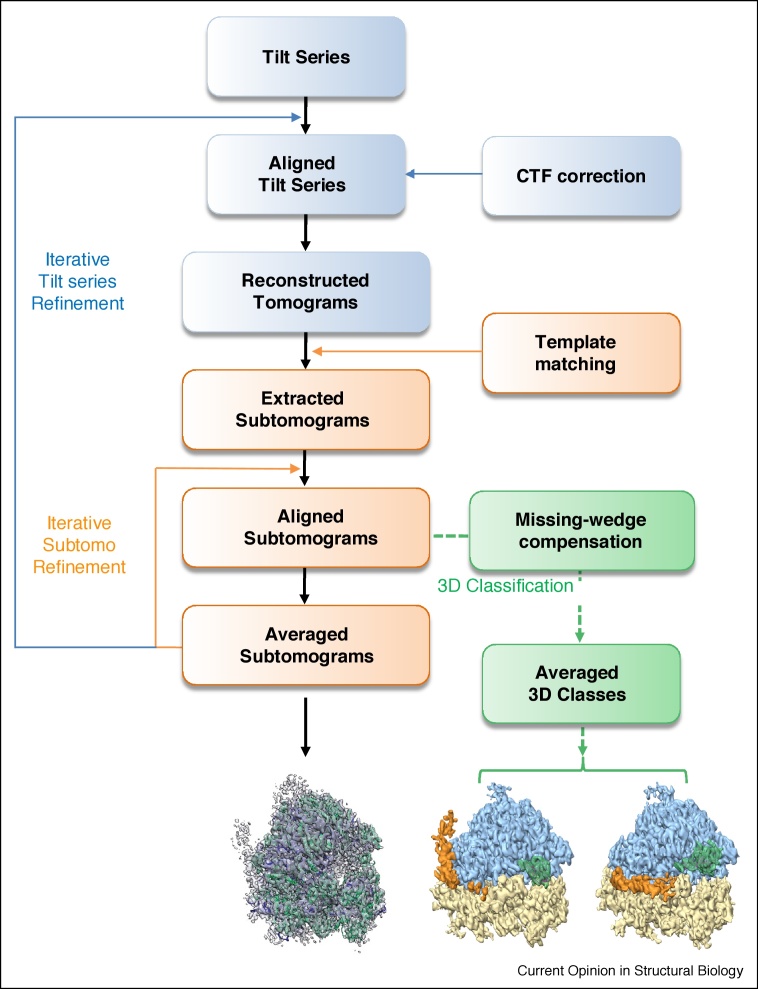
The overall workflow for cryoET subtomogram averaging and classification (cryoSTAC). The processes dealing with tilt series are in blue, subtomograms in orange, and 3D classification in green. The final structures at the bottom of the flowchart are yeast ribosomes.

The basic principles of cryoSTAC have been described in previous publications (for recent reviews see Refs. [3,8]). A typical cryoSTAC workflow is illustrated in Figure 1, with three main processing stages: the first stage is processing the tilt series (blue), the second stage is subtomogram averaging (orange), and the third involves 3D classification (green). A number of software packages are available for cryoSTAC processing, and Table 1 presents a comparison of the main features in commonly used packages, including PEET [9], EMAN2 [10,11], RELION [12], Dynamo [13], Jsubtomo [14], PyTom/AV3 [15,16], Protomo/i3 [17] and emClarity [18^••^].

**Table 1:** Comparison of features of major subtomogram averaging software packages

Major Software	PEET [9]	Eman2 [10,11]	RELION [12]	Dynamo [13]	Jsubtomo [14]	AV3/pyTOM [15,16]	Protomo/i3 [17]	emClarity [18]
Template Matching^a^	Manual	Auto-picking	No	Manual	Yes	Yes	Yes	Yes
3D CTF correction^b^	No	Per-particle	Per-particle	No	No	No	No	Yes
Missing-wedge compensation^c^	WMD^d^	Fourier Intensity	3D-Sampling	WMD	WMD	WMD	WMD	3D-Sampling
Tilt-series refinement	No	No	No	No	No	No	No	Yes
GPU support	No	No	No	Yes	No	No	No	Yes
GUI	Yes	Yes	Yes	Yes	No	Partial	No	No

a Method used for extracting subtomograms.

b 3D CTF correction algorithm such as implemented in NovaCTF [27^•^].

c Handling of missing-wedge in subtomogram alignment and classification.

d WMD: wedge masked differences.

The input data for a cryoSTAC workflow are a tilt series, that is, a series of cryoEM projection images recorded from the same specimen area with the specimen tilted over a range of angles, typically ±60°. There are several tilting schemes used for data collection, commonly the unidirectional (from −60 to +60), bidirectional (0 to −60, then 0 to +60), and more recently dose-symmetrical (alternating – and + tilts as the tilt angle increases, 0, −2, +2, −4, +4…) [19^•^]. The series of tilt images are then aligned, with or without the aid of fiducial beads such as gold particles added to the specimen, and the contrast transfer function (CTF) is determined and compensated [11,20]. A 3D volume (tomogram) can be reconstructed computationally from the aligned tilt series, commonly using a weighted back-projection algorithm as implemented in IMOD [21], although several other reconstruction methods are available [22, 23, 24]. The alignment of a tilt series can be further refined iteratively, using subtomograms as fiducial markers, as implemented in the newly developed emClarity [18^••^].

The 3D tomogram often contains multiple copies or instances of the complex of interest, which can be extracted, typically using a template-matching algorithm in which a known structure serves as a reference [4]. The angular orientation of the extracted subtomogram is refined iteratively in a way that is conceptually similar to SPA, but in 3D instead of 2D, and is used to generate an improved structure by averaging many copies of the object. Resolutions in the subnanometer range have been attained *in situ* [25^•^], and near-atomic *ex situ* [26^••^,27^•^].

These particles, as copies of the complex of interest in a tomogram, often vary in conformation and composition, and thus need to be separated using 3D classification. An important consideration during 3D classification is that tomograms are distorted along the z axis because of the missing data in reciprocal space resulting from the limited tilt range in data collection (also known as the ‘missing-wedge’). This must be taken into account in subtomogram classification, as the missing-wedge artifact tends to obscure real differences between subtomograms [4,9]. A simple approach to compensate for the missing-wedge effect is to apply the same subtomogram wedge to the reference average when these are compared with each other, as implemented in the binary wedge-masked difference (WMD) method [9,16]. More sophisticated approaches have been recently developed to correct for modulation of the CTF, such as Fourier intensity modulation [10] and full 3D sampling function [12,18^••^].

## Recent technical advances in cryoSTAC

In addition to the development of direct electron detectors, which have transformed all modalities of cryoEM, three main areas of recent technical advances have made cryoSTAC an exciting new method for *in situ* structure determination at subnanometer resolution: sample preparation, data collection, and image processing. Several new sample preparation methods have been developed to overcome the limitation of sample thickness (0.5 μm) for cryoEM imaging of bacteria and large eukaryotic cells *in situ*. These include mini-bacterial cell preparations [28], controlled bacterial lysis using a phage lysis gene [29], vitreous sectioning (CEMOVIS) [30], and, most significantly cryo-focused ion beam (cryoFIB) micromachining to create a 150–250 nm thick cell lamella, allowing access to any location inside of eukaryotic cells [31,32]. With regard to data acquisition, direct electron detectors, zero-loss imaging with an energy filter and phase plate imaging greatly enhance the SNR in extremely low-dose cryoET images [33]. Furthermore, tilt-series acquisition using the dose-symmetrical scheme ensures an optimal use of limited electron dose [19^•^]. Lastly, new algorithms have been developed and incorporated into cryoSTAC processing software (Table 1), including template matching for subtomogram extraction [16,17,18^••^], 3D-CTF for performing CTF corrections [10,18^••^,27^•^], tomoCPR for iteratively refining the tilt series alignment using subtomograms as fiducials [18^••^], use of a 3D-sampling function for improved ‘missing-wedge’ compensation [12,18^••^], and multiscale principal component analysis (PCA) for robust 3D classification [18^••^]. These technical improvements in cryoSTAC have had a significant impact on our understanding of the molecular mechanisms and biological functions of macromolecular assemblies, as exemplified in the cases discussed below.

## Successful applications

In recent years there have been numerous examples of cryoSTAC applications in various fields. These include membrane-associated and embedded complexes, such as Coat Protein Complex I (COPI) [34^•^,^35^], Nuclear Pore Complexes (NPCs) [36^•^,^37^], mitochondrial complex I and its supramolecular assemblies [38], mitochondrial ATP synthase [39,40], polysomes [41] and pore forming pneumolysin [42]; large assemblies in bacteria cells, such as chemotaxis signaling arrays [43,44^••^], the type IV pilus [45^••^], type III, type IV and Type VI secretion systems [46, 47, 48, 49, 50]; as well as bacteriophages [51,52] and viruses [26^••^,^53^, ^54^, ^55^,56^•^,57^••^]. For the majority of these systems, the resolution has been limited to 2–4 nm. Here, I focus on several recent studies that have achieved close to 1 nm resolution or better, and that have distinguished multiple functional states *in situ* using cryoSTAC. I show how the application of cryoSTAC has led to new insights into the function of macromolecular complexes that were not previously attainable.

### Molecular cryoSTAC at high resolution

**Figure 2:**
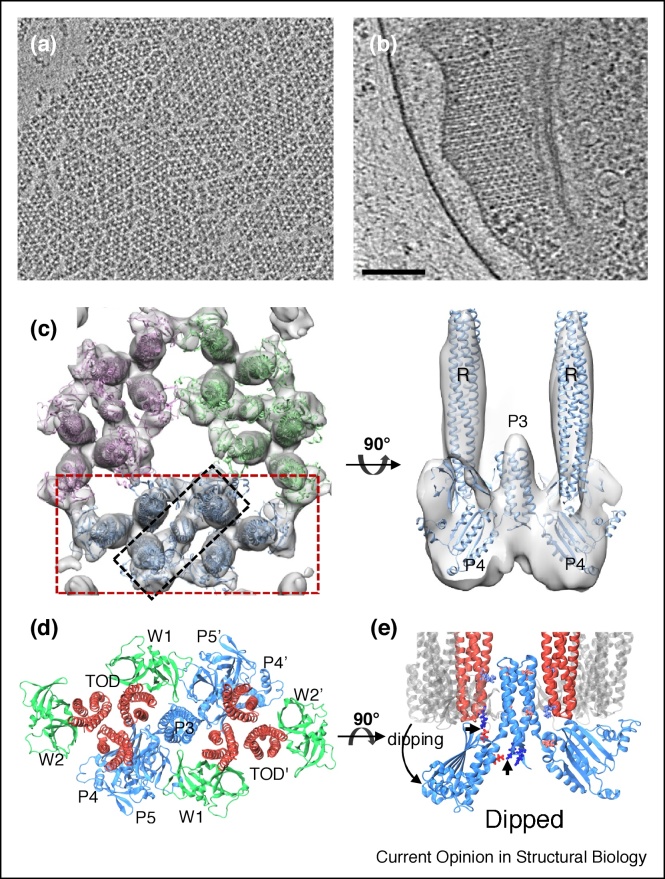
CryoSTAC of a reconstituted bacterial chemotaxis signaling array. **(a)** and **(b)** Tomographic slices of the *in vitro* reconstituted array of the chemotaxis core signaling complex (a) and of a native array in an *Escherichia coli* cell (b). Scale bar, 100 nm. **(c)** The density map of a threefold assembly unit by subtomogram averaging, with a core signaling complex boxed in red and a rotated view shown on the right corresponding to the black box. The receptor, P3 and P4 domains of Che A are labeled as ‘R’, ‘P3’ and ‘P4’, respectively. **(d)** Pseudo-atomic model of the core signaling complex, consisting of six chemoreceptor dimers (red, labeled ‘TOD’), one CheA dimer (blue, labeled ‘P3–P5’), and four CheW monomers (green, labeled ‘W1’ and ‘W2’). **(e)** CheA conformational dynamics with a dipping motion, determined by large scale MD simulation. Arrows point to the interacting amino acids in the dipped state [44^••^].

Biological complexes are often heterogeneous and not amenable to the SPA approach. In these cases, molecular cryoSTAC is an ideal approach to obtain their structures, given that the specimens are usually thin and contain many copies. By this approach, components of such biological systems can be isolated and reconstituted *in vitro* to reduce the complexity. An example of such a reconstituted system is the large and dynamical array comprised of bacterial chemotaxis core signaling complexes, which are responsible for monitoring the chemical environment and directing cell migration towards nutrient sources. Studies of native arrays using cryoSTAC have yielded a great deal of knowledge about the organization of the array [58,59], but with limited resolution. Using purified protein components, the signaling array can be reconstituted on a lipid monolayer, mimicking the structure in native bacterial cells (Figure 2a, b) [44^••^]. This relatively clean system with a large number of repeating units (∼3000 subtomograms) was amenable to cryoSTAC, from which the structure of the core signaling complex was determined at 11 Å resolution (Figure 2c). Guided by the structural details present in the subtomogram average, an atomic model of the whole signaling array revealed novel interfaces between the component proteins. In addition, molecular dynamics simulations revealed conformational dynamics of the core signaling complex (Figure 2d, e) [60].

**Figure 3:**
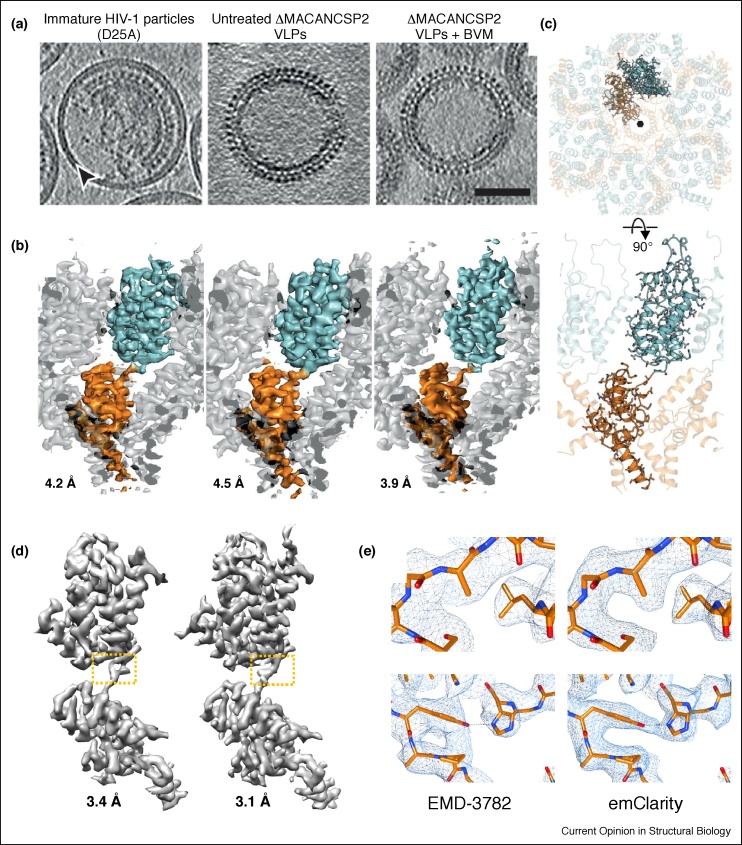
CryoSTAC of HIV-1 immature Gag particles. **(a)** Tomographic slices of immature HIV-1 particles (with D25A mutation), ΔMACANCSP2 VLPs, in the absence and presence of the maturation inhibitor bevirimat (BVM). Scale bar, 50 nm. **(b)** CA-SP1 densities from the samples shown in (a) by subtomogram averaging. One CA-SP1 monomer is highlighted, with the CA-NTD in cyan and the CA-CTD and SP1 in orange. **(c)** The refined atomic model. **(d)** An improved CA-SP1 density map at 3.1 Å resolution by emClarity (right), compared to the previous structure (left, EMD-3782). **(e)** Enlarged views of boxed area in (d) overlaid with a real-space refined model [18^••^,26^••^].

Many virus particles manifest icosahedral or helical symmetry and are amenable to high-resolution structure determination by SPA. Other viruses, such as HIV-1 are pleomorphic. Nevertheless, structure determination via cryoSTAC is still feasible because the pleomorphic capsids comprised many repeating units. By this approach Briggs and colleagues have determined some of the highest resolution subtomogram structures to date [26^••^,27^•^]. Examples of their impressive results include the structure of the asymmetric unit of the COPI coat protein assembled *in vitro* (∼40 000 subtomograms) at 9.2 Å resolution [35], the structure of immature virus-like Rous Sarcoma Virus Gag particles at 8 Å resolution [61]; the structure of Ebola virus nucleocapsid assemblies at 6.6 Å resolution [56^•^], and most significantly their work on HIV-1 immature and mature capsid assemblies [26^••^,57^••^]. The CA portion of immature HIV-1 Gag was initially solved by cryoSTAC to 8.8 Å [62], allowing unambiguous positioning of all α-helices. Optimizing data collection and image processing, as well as increasing the size of the dataset, led to a dramatic improvement of the structure to 3.9 Å resolution, which was further enhanced to 3.4 Å with 3D-CTF correction (Figure 3a–c) [26^••^,27^•^]. The near-atomic resolution structure reveals a network of interactions mediating immature HIV-1 assembly and a previously elusive SP1 six-helix bundle stabilized by a maturation inhibitor [26^••^]. In addition, structures of the CA hexamers and pentamers within mature capsids of native virions have been determined by cryoSTAC, which revealed a different pentamer organization compared to the previous X-ray crystal structures and how the quasi-hexagonal CA lattice flexes to form the variably curved capsid shell [57^••^].

The highest reported resolution of immature HIV-1 Gag particles determined by cryoSTAC is currently 3.1 Å (Figure 3d, e), owing to the development of emClarity [18^••^]. This new GPU-accelerated software features a novel iterative tilt-series refinement algorithm, a 3D-sampling function for missing-wedge compensation and a multiscale PCA classification (Figure 1, Table 1). These implementations have enabled a significant improvement in resolution and in 3D classification of different functional states in several test samples [18^••^]. The prospect of reaching atomic or near-atomic resolution by cryoSTAC and the ability to sort out multiple conformers have generated great interest in the cryoEM field.

### Cellular cryoSTAC for *in situ* structures and functional states

A large body of cellular cryoSTAC studies have been performed on a variety of intact bacterial cells because of their relatively small size. Because of the sparsity and complexity of the object of interest, these studies have resulted in mostly low resolution structures [45^••^,^46^, ^47^, ^48^, ^49^, ^50^]. One exception is the structure of the bacterial S-layer proteins, which has been determined to 7.4 Å by cryoSTAC [25^•^]. Docking of the X-ray structure into the subtomogram average resulted in a pseudo-atomic model of the S-layer, which revealed that the S-layer is porous and stabilized by multiple Ca^2+^ ions bound near the interfaces [25^•^].

**Figure 4:**
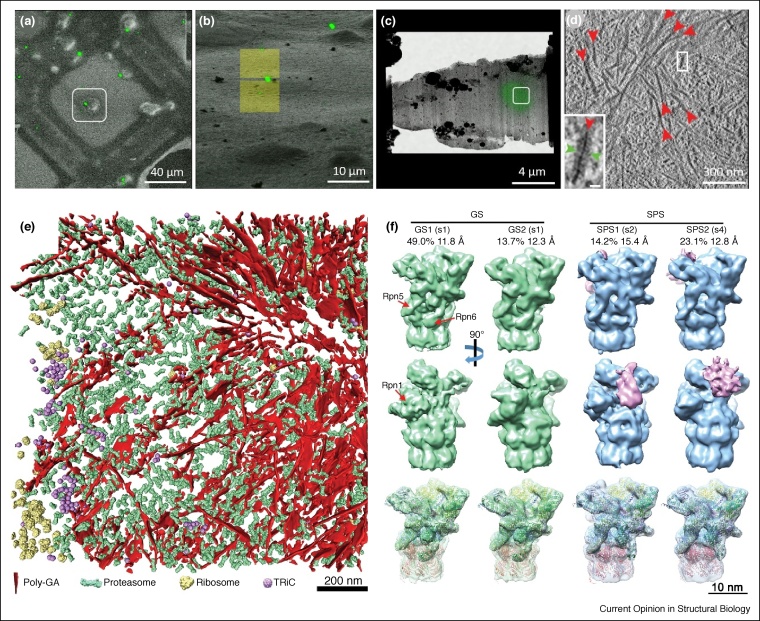
CryoSTAC of *in situ* Poly-GA aggregates with proteasomes recruitment in neurons. **(a)** and **(b)** Correlative cryo-light and cryoFIB/SEM of rat cortical neurons cultured on EM grids and transduced with (GA)175-GFP. SEM (a) and FIB (b) images were aligned and superimposed with the GFP signal from the cryo-LM image. **(c)** Cryo-TEM low magnification image of the lamella superimposed with the GFP signal. **(d)** A tomographic slice recorded in the area with GFP signal (white square in (c)). Red arrowheads mark a dense network of poly-GA-GFP. **(e)** 3D rendering of an aggregate within a neuron transduced with (GA)175-GFP showing different macromolecules found either within or at the periphery of the aggregate. **(f)** Subtomogram classification of 26S proteasomes reveals enrichment of substrate processing conformations. GS, ground state; SPS, substrate-processing state [7^••^].

Applications of cryoSTAC to large mammalian cells are much more challenging and usually require cryoFIB milling to produce cell lamellae of 150–250 nm in thickness for cryoEM imaging. *In situ* low resolution structures of the COPI coat [34^•^], NPC [63,64], adenovirus particles and microtubules [54], tripeptidyl peptidase II (TPPII) [65] and proteasomes [66] were determined using cryoFIB and cryoSTAC. Most recently, Baumeister’s lab investigated structures of protein aggregates inside neurons, in particular polyglutamine (polyQ)-expanded huntingtin exon 1 and poly-Gly-Ala (poly-GA) aggregates. For this purpose cryo-correlative light and electron microscopy (cryoCLEM) were used to target polyQ and poly-GA inclusions for cryoFIB milling [7^••^,67^•^]. They showed that PolyQ inclusions in neurons consist of amyloid-like fibrils, interact with and deform ER membranes and alter ER organization and dynamics without harbouring a significant amount of 26S proteasomes [67^•^]. In contrast, Poly-GA aggregates consist of more densely packed planar twisted ribbons that recruit numerous 26S proteasome complexes (Figure 4) [7^••^]. CryoSTAC analysis of recruited 26S proteasomes, in both ground-processing and substrate-processing states, revealed an enrichment of substrate processing conformations and proteasome stalling upon interaction with poly-GA aggregates. The study suggests that poly-GA aggregates may compromise neuronal proteostasis by sequestering and functionally impairing a large fraction of cellular proteasomes. As exemplified in these studies, cellular cryoSTAC combined with 3D classification revealed different conformational states of protein complexes. Their spatial distributions could be mapped in native cells, which changed in response to various perturbations, thus opening a new frontier in structural cell biology.

## Future perspective

Compared to cryoEM SPA, cryoSTAC is still in its early stages. However, there are examples in which near-atomic resolution structures have been determined, and multiple functional states have been delineated *in situ*, allowing a direct connection between cellular function and the structure of protein complexes. The greatest strength of cryoSTAC lies in *in situ* structure determination with 3D classification in native systems. It holds the potential to provide cellular landscapes of macromolecular complexes in near-atomic details with their spatial coordinates (or molecular census) [68]. Yet, the method is limited, as many proteins are too small and too rare, falling below the detection limit. In addition, extreme crowding within the cytoplasm greatly impedes the ability to distinguish individual proteins and protein complexes.

Some of the latest technologies, including direct electron detectors, zero-loss imaging with an energy filter, phase plate imaging to enhance contrast, and cryoFIB milling for access to the interior of thick cells, have proven to be extremely valuable for deriving higher resolution structures by cryoSTAC approaches. There are still many avenues for further improvement and optimization, and many applications to explore. In cryoSTAC processing, algorithms need to be developed to properly handle cryoET data collected using a phase plate. For *in situ* sample preparation, cryoFIB milling of frozen-hydrated samples is by far the best method, but current applications are limited to samples that are less than 5 μm thick, where proper vitrification can be achieved via plunge freezing. A routine cryoFIB lift-out [69] procedure would greatly facilitate cryoSTAC studies of thicker mammalian cells and even tissues that are high-pressure frozen. CryoCLEM is critical for targeting areas of interest in cryoFIB, but currently its precision is limited to a few hundred nanometers within the imaging plane and much worse in the Z direction. Further enhancement in super resolution cryoCLEM could potentially allow correlations at the single molecule level, which, in combination with improved template matching, will make the localization and identification of macromolecules in 3D tomograms almost entirely unambiguous. And finally, with further development in time-resolved cryoEM [70], we can begin to capture changes of molecular complexes in conformation and localization upon perturbation. By revealing the structures and atlas of macromolecular complexes *in situ* and in time, cryoET and cryoSTAC approaches will have an immense impact on our mechanistic understanding of biological systems, in normal and pathological physiology.

## Conflict of interest statement

Nothing declared.

## References and recommended reading

Papers of particular interest, published within the period of review, have been highlighted as:

• of special interest
•• of outstanding interest
